# Garlic Essential Oil as Promising Option for the Treatment of Acute Campylobacteriosis—Results from a Preclinical Placebo-Controlled Intervention Study

**DOI:** 10.3390/microorganisms9061140

**Published:** 2021-05-25

**Authors:** Markus M. Heimesaat, Soraya Mousavi, Dennis Weschka, Stefan Bereswill

**Affiliations:** Gastrointestinal Microbiology Research Group, Institute of Microbiology, Infectious Diseases and Immunology, Charité-Universitätsmedizin Berlin, Corporate Member of Freie Universität Berlin, Humboldt-Universität zu Berlin, and Berlin Institute of Health, 12203 Berlin, Germany; dennis.weschka@charite.de (D.W.); stefan.bereswill@charite.de (S.B.)

**Keywords:** garlic essential oil, enteropathogenic infection, *Campylobacter jejuni*, immune-modulatory effects, secondary abiotic IL-10^-/-^ mice, acute campylobacteriosis model, host-pathogen interaction, preclinical placebo-controlled intervention study, natural antibiotics-independent compounds

## Abstract

Since human infections with *Campylobacter jejuni* including antibiotic-resistant strains are rising worldwide, natural compounds might constitute promising antibiotics-independent treatment options for campylobacteriosis. Since the health-beneficial properties of garlic have been known for centuries, we here surveyed the antimicrobial and immune-modulatory effects of garlic essential oil (EO) in acute experimental campylobacteriosis. Therefore, secondary abiotic IL-10^-/-^ mice were orally infected with *C. jejuni* strain 81-176 and garlic-EO treatment via the drinking water was initiated on day 2 post-infection. Mice from the garlic-EO group displayed less severe clinical signs of acute campylobacteriosis as compared to placebo counterparts that were associated with lower ileal *C. jejuni* burdens on day 6 post-infection. Furthermore, when compared to placebo application, garlic-EO treatment resulted in alleviated colonic epithelia cell apoptosis, in less pronounced *C. jejuni* induced immune cell responses in the large intestines, in dampened pro-inflammatory mediator secretion in intestinal and extra-intestinal compartments, and, finally, in less frequent translocation of viable pathogens from the intestines to distinct organs. Given its potent immune-modulatory and disease-alleviating effects as shown in our actual preclinical placebo-controlled intervention study, we conclude that garlic-EO may be considered as promising adjunct treatment option for acute campylobacteriosis in humans.

## 1. Introduction

Human infections with enteropathogens such as *Campylobacter jejuni* are progressively rising all over the globe, causing tremendous health burdens with serious socioeconomic consequences [1]. The Gram-negative bacteria are commonly found as commensal members of the gut microbiota in many avian hosts, including poultry such as chicken and turkeys [2]. In the majority of events, contaminations of meat products occur during the slaughter processes, facilitating access of the *C. jejuni* bacteria to the food chain [3]. Humans may become infected upon ingestion of undercooked or even raw meat products, milk or surface waters that are contaminated with *C. jejuni* [2]. The highly motile bacteria express a broad range of virulence factors involved in cell adhesion and invasion and induce innate and adaptive host immune responses limiting bacterial dissemination on one hand but at the expense of resultant tissue damage on the other [2,4]. Within two to five days post bacterial ingestion, individuals might complain about clinical signs such as common discomfort with headache and even fever, and about gastrointestinal malaise as characterized by nausea, vomiting, abdominal pain, and watery or even bloody diarrhea with mucous and inflammatory discharges [2,3,4]. Immune-competent individuals may require a symptomatic treatment regimen consisting of antipyretics and substitution of fluids and minerals, whereas antibiotic treatment, however, may be indicated in severe cases affecting immune-compromised patients, for instance [5,6]. Whereas the patients might suffer from *C. jejuni* induced clinical signs for up to a week, symptoms usually completely resolve within 10–14 days post-infection (p.i.) without residues [2,6]. Very rarely, however, post-infectious autoimmune diseases including Guillain-Barré syndrome, reactive arthritis or intestinal morbidities such as irritable bowel syndrome, celiac disease and chronic inflammatory bowel diseases may evolve [2,6,7,8,9,10]. The *C. jejuni* endotoxin lipooligosaccharide (LOS) derived from the bacterial cell wall constitutes a key molecule in inducing pro-inflammatory host immune responses leading to apoptosis, compromised intestinal epithelial barrier integrity and subsequent malabsorption [11]. The *C. jejuni*-LOS is not only responsible for the severity of infectious enteritis, but also for the post-infectious sequelae depending on its sialylation status [12]. Given the emergence of multi-drug resistance among bacteria including *C. jejuni*, natural compounds might constitute promising antibiotics-independent treatment options for campylobacteriosis.

Garlic (*Allium Sativum* L.) belongs to the onion family (*Alliaceae*), constitutes one of the most frequently used herbs in the world, and contains more than 200 biologically active ingredients [13]. The usage of the natural compound originating from central Asia in folk medicine dates back to 2600 BC [14,15]. To date, garlic and its derivatives have been found to possess a broad diversity of health-beneficial properties [16], including anti-oxidant [17,18,19], anti-microbial [20,21,22,23,24], and immune-modulatory effects as shown both in vitro and in vivo [25,26,27,28,29]. Therefore, garlic and its derivatives are considered as promising adjunct treatment option for various diseases including infectious enteritis [30]. Furthermore, a previous in vitro study applying garlic essential oil (EO) revealed potent antimicrobial effects directed against *C. jejuni* ATCC 33291 strain [31].

This prompted us to perform a preclinical placebo-controlled intervention study assessing potential anti-pathogenic and immune-modulatory effects of garlic-EO in acute experimental campylobacteriosis applying the secondary abiotic interleukin-10 deficient (IL-10^-/-^) mouse infection and inflammation model [32]. Following microbiota depletion by antibiotic pretreatment, IL-10^-/-^ mice develop acute campylobacteriosis characterized by wasting symptoms and bloody diarrhea, by acute enterocolitis with colonic epithelial cell apoptosis and by enhanced pro-inflammatory immune responses in the intestinal tract, in extra-intestinal organs and even systemically within a week post oral *C. jejuni* infection [4,32,33]. Furthermore, the pronounced *C. jejuni* induced immune responses in those animals have been shown to be due to bacterial LOS-induced Toll-like receptor-4 (TLR-4) signaling [32]. This acute murine *C. jejuni* infection and inflammation model which depends on bacterial motility, adhesion and invasion has been proven highly reliable in assessing the disease-alleviating properties of non-toxic natural compounds during acute campylobacteriosis as recently shown for cardamom-EO [34] and clove-EO [35]. Here, we assessed the gastrointestinal pathogen loads, the clinical outcome, the microscopic inflammatory changes and immune cell responses in the colon, the pro-inflammatory mediator secretion in the intestinal tract and beyond, and the bacterial translocation to extra-intestinal organs following garlic-EO as compared to placebo treatment of *C. jejuni* infected secondary abiotic IL-10^-/-^ mice.

## 2. Materials and Methods

### 2.1. Ethical Statement

Mouse experiments were approved by the local ethical committee for animal experiments (“Landesamt für Gesundheit und Soziales”, LaGeSo, Berlin; registration number G0104/19) and carried out according to the European guidelines for animal welfare (2010/63/EU) that was monitored daily by clinical surveyance of each mouse.

### 2.2. Secondary Abiotic IL-10^-/-^ Mice

In the Forschungsinstitute für Experimentelle Medizin, Charité-Universitätsmedizin Berlin, Germany, IL-10^-/-^ mice (C57BL/6j background) were raised and housed in cages with filter tops under standard conditions within an experimental semi-barrier facility. Mice had free access to standard chow (food pellets: ssniff R/M-H, V1534-300, Sniff, Soest, Germany) and to autoclaved water. Three-week-old female and male mice were treated with an antibiotic cocktail for commensal gut microbiota depletion as stated elsewhere [36,37]. In brief, following transfer to sterile cages mice received ciprofloxacin (200 mg/L; Fresenius Kabi, Bad Homburg, Germany), metronidazole (1 g/L; B. Braun, Melsungen, Germany), imipenem (250 mg/L; Fresenius Kabi, Bad Homburg, Germany), ampicillin plus sulbactam (1 g/L; Dr. Friedrich Eberth Arzneimittel, Ursensollen, Germany), and furthermore, vancomycin (500 mg/L; Hikma Pharmaceuticals, London, UK) via the drinking water for eight weeks (*ad libitum*). Secondary abiotic IL-10^-/-^ mice were handled under strict aseptic conditions. Three days prior *C. jejuni* infection, the animals received autoclaved tap water.

### 2.3. C. jejuni Infection and Treatment Regimens

Following thawing from frozen stocks, *C. jejuni* strain 81-176 was grown on selective karmali agar plates and on Columbia agar (with 5% sheep blood, both from Oxoid, Wesel, Germany). On days 0 and 1, sex- and age-matched 4-month-old littermate secondary abiotic IL-10^-/-^ mice were perorally challenged with 10^9^ colony forming units (CFU) of the pathogen by gavage. Treatment with garlic-EO (purchased from Sigma-Aldrich, Munich, Germany) was initiated on day 2 p.i. and performed until the end of the observation period (i.e., day 6 p.i.). Therefore, the natural compound was dissolved in sterile phosphate buffered saline (PBS, Thermo Fisher Scientific, Waltham, MA, USA) and 0.05% carboxymethyl cellulose and then applied to autoclaved tap water (final concentration of 1 g/L; *ad libitum*; daily dose of 200 mg/kg body weight given a mean body weight of 20 g and an average daily drinking volume of 5 mL). The placebo control mice received respective solution without the natural compound.

### 2.4. Pathogenic Colonization and Translocation

The pathogenic loads were surveyed in fecal samples at defined time points p.i., and upon necropsy in luminal samples derived from the colon, ileum, duodenum and stomach and additionally, in ex vivo biopsies taken from mesenteric lymph nodes (MLN), spleen, lungs, liver, and kidneys by culture as reported earlier [36,38]. Briefly, by using sterile pestles respective samples were homogenized in sterile PBS (Thermo Fisher Scientific, Waltham, MA, USA). Serial dilutions were then streaked onto karmali agar and Columbia agar (supplemented with 5% sheep blood; both from Oxoid, Wesel, Germany) and incubated in a jar containing CampyGen gas packs (Oxoid, Wesel, Germany) under microaerophilic conditions at 37 °C for at least 48 h. In order to survey systemic translocation, cardiac blood (0.1 mL) was directly plated onto Columbia agar (supplemented with 5% sheep blood) and incubated likewise. The detection limit of viable pathogens was 100 CFU per g.

### 2.5. Clinical Conditions

Immediately before and at defined time points p.i., we quantitatively surveyed the clinical conditions of mice applying clinical scores (maximum 12 points), addressing the abundance of blood in feces (0: no blood; 2: microscopic detection of blood by the Guajac method using Haemoccult, Beckman Coulter/PCD, Germany; 4: macroscopic blood visible), the stool consistency (0: formed feces; 2: pasty feces; 4: liquid feces) and the clinical aspect (i.e., wasting symptoms; 0: normal; 1: ruffled fur; 2: less locomotion; 3: isolation; 4: severely compromised locomotion, pre-final aspect), as reported previously [39].

### 2.6. Sampling Procedures

On the day of necropsy (i.e., day 6 p.i.), mice were sacrificed by CO_2_ asphyxiation. Cardiac blood, ex vivo biopsies from MLN, spleen, lungs, liver, kidneys, ileum, and colon, as well as luminal samples from colon, ileum, duodenum and stomach were collected under sterile conditions. From each mouse, large intestinal ex vivo biopsies were derived in parallel for subsequent microbiological, immunohistopathological and immunological analyses.

### 2.7. Histopathology

Histopathological analyses were performed in colonic ex vivo biopsies following immediate fixation (5% formalin) and paraffin embedding. Upon staining with hematoxylin and eosin (H&E), sections (5 µm) were examined regarding histopathological changes in the large intestines by light microscopy (100× magnification), which were quantitatively assessed with histopathological scores as described earlier [40]. In brief, score 1, minimal inflammatory cell infiltrates in the mucosa with intact epithelium. Score 2, mild inflammatory cell infiltrates in the mucosa and submucosa with mild hyperplasia and mild goblet cell loss. Score 3, moderate inflammatory cell infiltrates in the mucosa with moderate goblet cell loss. Score 4 marked inflammatory cell infiltration into the mucosa and submucosa with marked goblet cell loss, multiple crypt abscesses, and crypt loss.

### 2.8. In Situ Immunohistochemistry

By applying quantitative in situ immunohistochemistry, colonic ex vivo biopsies (immediately fixed in 5% formalin and embedded in paraffin) were examined regarding abundances of F4/80^+^ macrophages/monocytes (no. 14-4801, clone BM8, eBioscience, San Diego, CA, USA; 1:50), CD3^+^ T lymphocytes (no. N1580, Dako, Glostrup, Denmark; 1:10) and cleaved caspase-3^+^ apoptotic epithelial cells (Casp3; Asp175, Cell Signaling, Beverly, MA, USA; 1:200) as stated earlier f [41,42]. Positively stained cells were enumerated by an independent investigator using light microscopy. The average number of respective positively stained cells in each sample was determined within at least six high power fields (HPF, 0.287 mm^2^, 400× magnification).

### 2.9. Pro-Inflammatory Mediators

Intestinal samples that had been collected from the ileum and colon (longitudinally cut strips of approximately 1 cm^2^, washed in PBS) and ex vivo biopsies derived from MLN; 3 nodes), the liver (approximately 1 cm^3^), the kidney (one half after the longitudinal cut), the lung (one half), and the spleen (one third) were transferred to 24-flat-bottom well culture plates (Thermo Fisher Scientific, Waltham, MA, USA) supplemented with 500 µL serum-free RPMI 1640 medium (Thermo Fisher Scientific, Waltham, MA, USA) plus penicillin (100 µg/mL) and streptomycin (100 µg/mL; Biochrom, Berlin, Germany). After an 18-h incubation period at 37 °C, respective culture supernatants and serum samples were tested for monocyte chemoattractant protein-1 (MCP-1), interleukin-6 (IL-6), interferon-γ (IFN-γ), and tumor necrosis factor-α (TNF-α), by applying the Mouse Inflammation Cytometric Bead Assay (CBA; BD Biosciences, Germany) in a BD FACSCanto II flow cytometer (BD Biosciences). The Griess reaction was used for nitric oxide measurements as described earlier [39].

### 2.10. Statistical Analyses

Medians and significance levels were assessed by using GraphPad Prism (version 8; San Diego, CA, USA). Normalization of data was surveyed by the Anderson-Darling test. The Mann-Whitney test was applied for pairwise comparisons of not normally distributed data. For multiple comparisons, the one-way ANOVA test with Tukey post-correction (for normally distributed data) and the Kruskal-Wallis test with Dunn’s post-correction (for not normally distributed data) were performed. Two-sided probability (*p*) values ≤ 0.05 were considered significant. Definite outliers were removed after having been identified by the Grubb’s test (α = 0.001). Data were pooled from four independent experiments.

## 3. Results

### 3.1. Impact of Garlic-Essential Oil (EO) Treatment on C. jejuni Colonization of the Gastrointestinal Tract in IL-10^-/-^ Mice

**Figure 1:**
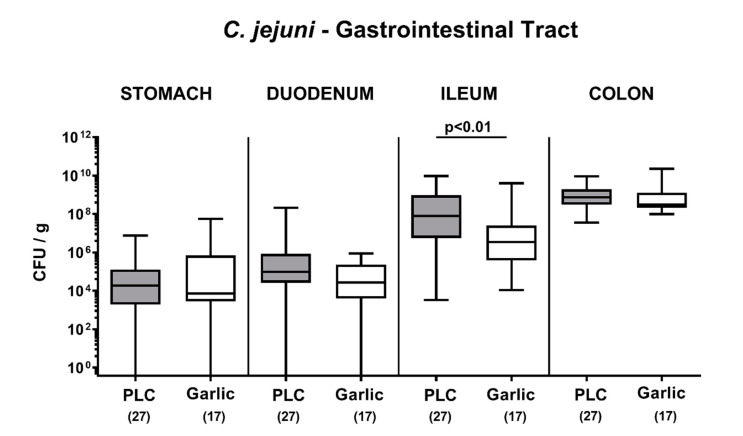
Pathogenic colonization of the gastrointestinal tract upon garlic essential oil treatment of *C. jejuni* infected IL-10^-/-^ mice. Secondary abiotic IL-10^-/-^ mice were orally challenged with *C. jejuni* strain 81-176 on days 0 and 1 followed by treatment with either garlic essential oil (white boxes) or placebo (PLC, gray boxes) via the drinking water starting on day 2 post-infection. On day 6 post-infection, *C. jejuni* was quantitated in defined gastrointestinal luminal samples by culture (colony forming units per gram; CFU/g). Box plots (indicating the 25th and the 75th percentiles), whiskers (indicating maximum and minimum values), medians (black bar inside box), significance levels (*p* values) determined by the Mann-Whitney U test and the total number of mice (in parentheses) are shown (pooled data from four independent experiments).

We first addressed whether garlic-EO treatment of *C. jejuni* infected secondary abiotic IL-10^-/-^ mice interfered with pathogenic colonization efficacies within the gastrointestinal tract. Therefore, luminal samples were derived from distinct gastrointestinal compartment upon necropsy. Our cultural analyses revealed comparable *C. jejuni* loads in the stomach, duodenum and colon in mice from both cohorts on day 6 p.i., whereas garlic-EO treated mice harbored approximately 1.5 log orders of magnitude lower pathogenic cell numbers in their ileum as compared to placebo control mice (*p* < 0.01; Figure 1). Hence, garlic-EO treatment was associated with lower ileal *C. jejuni* burdens.

### 3.2. Clinical Outcome upon Garlic-EO Treatment of C. jejuni Infected IL-10^-/-^ Mice

**Figure 2:**
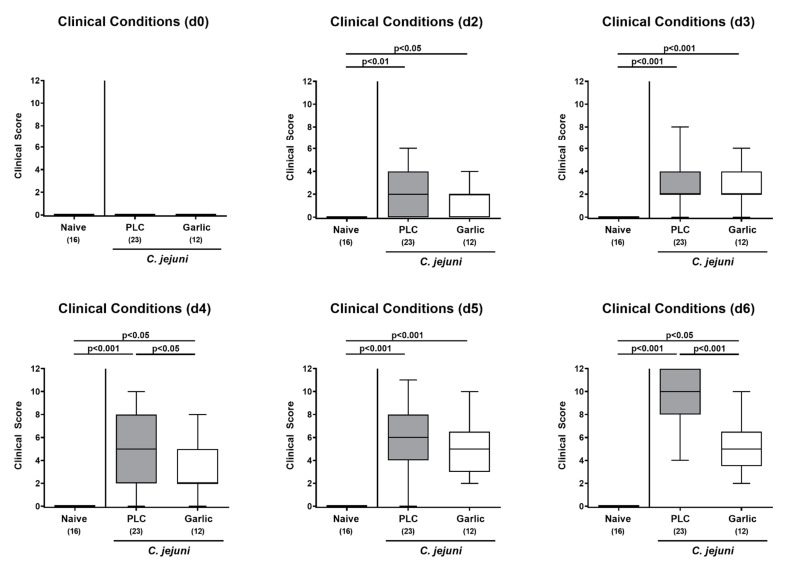
Time course of clinical conditions upon garlic essential oil treatment of *C. jejuni* infected IL-10^-/-^ mice. Secondary abiotic IL-10^-/-^ mice were orally challenged with *C. jejuni* strain 81-176 on day (d) 0 and d1 followed by treatment with either garlic essential oil (white boxes) or placebo (PLC, gray boxes) via the drinking water starting on d2 post-infection. The clinical conditions of mice were quantitated by using distinct clinical scores (see methods). Naive mice (hatched boxes) served as non-infected and untreated cohort. Box plots (indicating the 25th and the 75th percentiles), whiskers (indicating maximum and minimum values), medians (black bar inside box), significance levels (*p* values) determined by the Kruskal-Wallis test and Dunn’s post-correction and the number of analyzed animals (in parentheses) are shown (pooled data from four independent experiments).

We then addressed whether garlic-EO treatment would have a beneficial effect on the clinical conditions in *C. jejuni* infected mice and quantitated pathogen-induced disease by using clinical scores. Whereas mice from the placebo cohort were suffering from acute campylobacteriosis on day 6 p.i. as indicated by wasting symptoms and bloody diarrhea (Figure 2), garlic-EO treated mice exhibited less pronounced clinical scores for the overall clinical outcome (*p* < 0.001; Figure 2). Moreover, earlier in the course of disease (namely, on day 4 p.i.), the clinical scores were lower in garlic-EO as compared to placebo treated mice (*p* < 0.05; Figure 2). Hence, garlic-EO treatment was associated with alleviated clinical signs of acute campylobacteriosis.

### 3.3. Microscopic Inflammatory Sequelae upon Garlic-EO Treatment of C. jejuni Infected IL-10^-/-^ Mice

**Figure 3:**
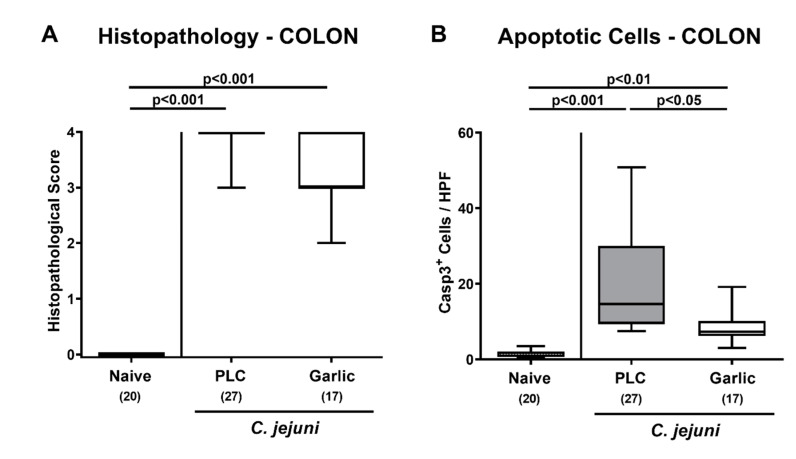
Microscopic inflammatory sequelae upon garlic essential oil treatment of *C. jejuni* infected IL-10^-/-^ mice. Secondary abiotic IL-10^-/-^ mice were orally challenged with *C. jejuni* strain 81-176 on days 0 and 1 followed by treatment with either garlic essential oil (white boxes) or placebo (PLC, gray boxes) via the drinking water starting on day 2 post-infection. On day 6 post-infection, (**A**) microscopic inflammatory changes were assessed in colonic ex vivo biopsies applying histopathological scores. Furthermore, (**B**) the mean numbers of apoptotic colonic epithelial cells out of six high power fields (HPF, 400× magnification) were enumerated microscopically in paraffin sections that had been stained with an antibody against cleaved caspase3 (Casp3^+^). Naive mice (hatched boxes) served as non-infected and untreated cohort. Box plots (indicating the 25th and the 75th percentiles), whiskers (indicating maximum and minimum values), medians (black bar inside box), significance levels (*p* values) determined by the Kruskal-Wallis test and Dunn’s post-correction and the total number of mice (in parentheses) are shown (pooled data from four independent experiments).

We then asked whether the disease-alleviating effects of garlic-EO could also be observed on the microscopic level. Therefore, we surveyed histopathological changes in the infected large intestines by using histopathological scores. On day 6 p.i., mice from either cohort displayed elevated histopathological scores (*p* < 0.001 versus naive; Figure 3A), but with a trend towards lower scores in garlic-EO as compared to placebo treated animals (not significant (n.s.) due to high standard deviation in the former; Figure 3A). Since apoptosis is commonly used for the microscopic grading of inflammatory morbidities of the intestinal tract including acute campylobacteriosis [36], we performed quantitative in situ immunohistochemical analyses of colonic paraffin sections. *C. jejuni* infected mice displayed higher numbers of cleaved caspase-3^+^ cells in colonic epithelia (*p* < 0.01–0.001; Figure 3B), but with more than 50% lower median numbers in garlic-EO as compared to placebo treated mice on day 6 p.i. (*p* < 0.05; Figure 3B). Hence, garlic-EO treatment of *C. jejuni* infected mice was associated with dampened colonic epithelial cell apoptosis.

### 3.4. Garlic-EO Treatment Dampens Colonic Immune Cell Responses in C. jejuni Infected IL-10^-/-^ Mice

**Figure 4:**
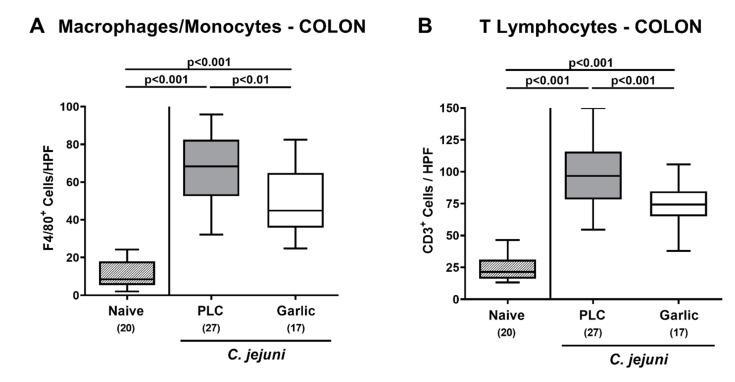
Colonic immune cell responses upon garlic essential oil treatment of *C. jejuni* infected IL-10^-/-^ mice. Secondary abiotic IL-10^-/-^ mice were orally challenged with *C. jejuni* strain 81-176 on days 0 and 1 followed by treatment with either garlic essential oil (white boxes) or placebo (PLC, gray boxes) via the drinking water starting on day 2 post-infection. On day 6 post-infection, the mean numbers of (**A**) macrophages and monocytes (positive for F4/80) and of (**B**) T lymphocytes (positive for CD3) out of six high power fields (HPF, 400× magnification) were enumerated microscopically in paraffin sections following immunohistochemical staining. Naive mice (hatched boxes) served as non-infected and untreated cohort. Box plots (indicating the 25th and the 75th percentiles), whiskers (indicating maximum and minimum values), medians (black bar inside box), significance levels (*p* values) determined by the one-way ANOVA with Tukey post-correction and the Kruskal-Wallis test and Dunn’s post-correction, and the total number of mice (in parentheses) are shown (pooled data from four independent experiments).

We then asked if garlic-EO treatment might interfere with *C. jejuni* induced immune cell responses. To test this, we stained colonic paraffin sections with antibodies directed against F4/80 and CD3 in order to quantitate macrophages/monocytes and T lymphocytes representing innate and adaptive immune cell populations involved in *C. jejuni* induced immunopathology, respectively [4]. On day 6 p.i., mice from either cohort displayed markedly increased numbers of macrophages/monocytes and of T lymphocytes in their colonic mucosa and lamina propria (*p* < 0.001 versus naive; Figure 4). These increases were, however, far less pronounced in garlic-EO as compared to placebo treated mice (*p* < 0.01–0.001; Figure 4). Hence, garlic-EO treatment was associated with less pronounced *C. jejuni* induced immune responses in the large intestines.

### 3.5. Reduced Pro-Inflammatory Mediator Secretion in the Intestinal Tract upon Garlic-EO Treatment of C. jejuni Infected IL-10^-/-^ Mice

**Figure 5:**
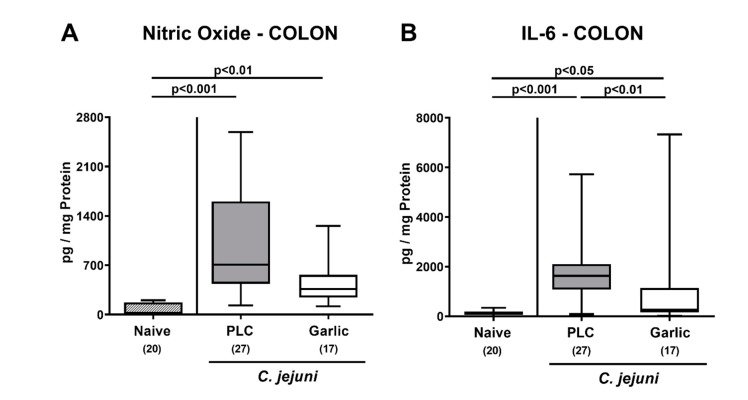
Pro-inflammatory mediator secretion in the colon upon garlic essential oil treatment of *C. jejuni* infected IL-10^-/-^ mice. Secondary abiotic IL-10^-/-^ mice were orally challenged with *C. jejuni* strain 81–176 on days 0 and 1 followed by treatment with either garlic essential oil (white boxes) or placebo (PLC, gray boxes) via the drinking water starting on day 2 post-infection. (**A**) Nitric oxide and (**B**) IL-6 concentrations were determined in culture supernatants of colonic explants taken on day 6 post-infection. Naive mice (hatched boxes) served as non-infected and untreated cohort. Box plots (indicating the 25th and the 75th percentiles), whiskers (indicating maximum and minimum values), medians (black bar inside box), significance levels (*p* values) determined by the Kruskal-Wallis test and Dunn’s post-correction and the total number of mice (in parentheses) are shown (pooled data from four independent experiments).

We then surveyed the impact of garlic-EO treatment on pro-inflammatory mediator secretion in distinct parts of the intestinal tract during acute murine campylobacteriosis. On day 6 p.i., enhanced nitric oxide and IL-6 secretion could be assessed in the colon of both, placebo and garlic-EO treated mice (*p* < 0.05–0.001; Figure 5), but with lower IL-6 concentrations measured in the latter versus the former (*p* < 0.01; Figure 5B).

**Figure 6:**
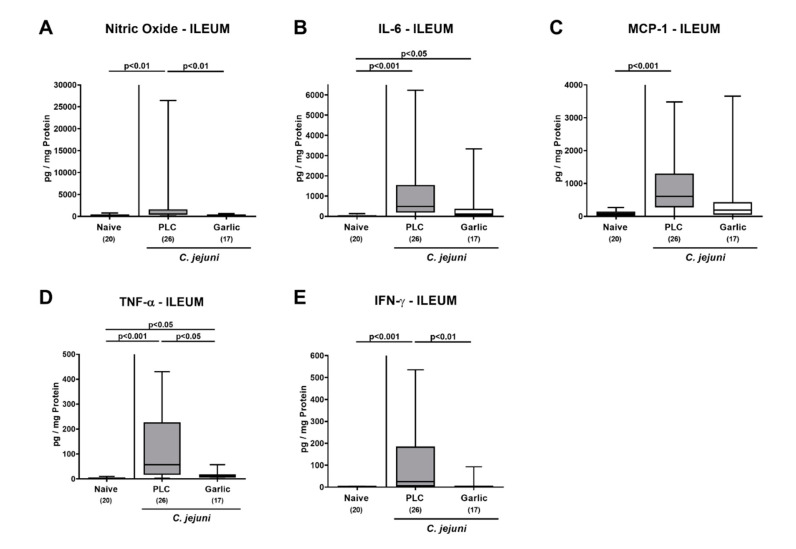
Pro-inflammatory mediator secretion in the ileum upon garlic essential oil treatment of *C. jejuni* infected IL-10^-/-^ mice. Secondary abiotic IL-10^-/-^ mice were orally challenged with *C. jejuni* strain 81–176 on days 0 and 1 followed by treatment with either garlic essential oil (white boxes) or placebo (PLC, gray boxes) via the drinking water starting on day 2 post-infection. (**A**) Nitric oxide, (**B**) IL-6, (**C**) MCP-1, (**D**) TNF-α and (**E**) IFN-γ concentrations were determined in culture supernatants of ileal explants taken on day 6 post-infection. Naive mice (hatched boxes) served as non-infected and untreated cohort. Box plots (indicating the 25th and the 75th percentiles), whiskers (indicating maximum and minimum values), medians (black bar inside box), significance levels (*p* values) determined by the Kruskal-Wallis test and Dunn’s post-correction and the total number of mice (in parentheses) are shown (pooled data from four independent experiments). Outliers were excluded after identification by the Grubb’s test (α = 0.001).

In the ileum, increased nitric oxide, MCP-1 and IFN-γ concentrations were observed in placebo as opposed to garlic-EO treated mice on day 6 p.i. (*p* < 0.01–0.001 versus naive; Figure 6A,C,E). Furthermore, *C. jejuni* infection resulted in elevated ileal IL-6 and TNF-α concentrations (*p* < 0.05–0.001 versus naive; Figure 6B,D). In case of ileal TNF-α, concentrations were lower in the garlic-EO as compared to the placebo cohort on day 6 p.i. (*p* < 0.05; Figure 6D), whereas a trend towards lower IL-6 levels could be observed in ileal samples taken from the former versus the latter (n.s. due to high standard deviations; Figure 6B).

**Figure 7:**
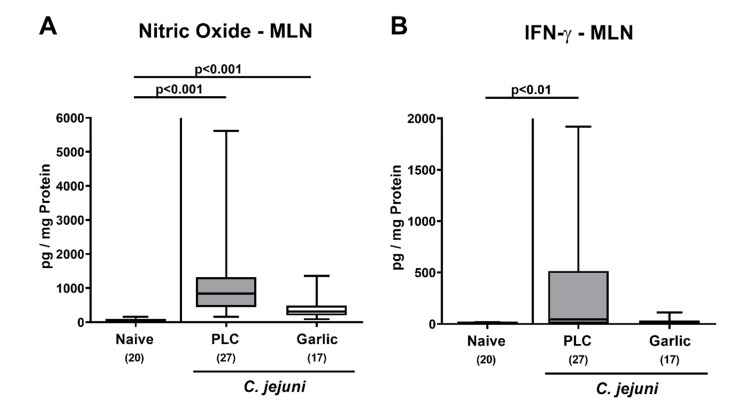
Pro-inflammatory mediator secretion in mesenteric lymph nodes upon garlic essential oil treatment of *C. jejuni* infected IL-10^-/-^ mice. Secondary abiotic IL-10^-/-^ mice were orally challenged with *C. jejuni* strain 81-176 on days 0 and 1 followed by treatment with either garlic essential oil (white boxes) or placebo (PLC, gray boxes) via the drinking water starting on day 2 post-infection. (**A**) Nitric oxide and (**B**) IFN-γ concentrations were determined in culture supernatants of mesenteric lymph nodes (MLN) explants taken on day 6 post-infection. Naive mice (hatched boxes) served as non-infected and untreated cohort. Box plots (indicating the 25th and the 75th percentiles), whiskers (indicating maximum and minimum values), medians (black bar inside box), significance levels (*p* values) determined by the Kruskal-Wallis test and Dunn’s post-correction and the total number of mice (in parentheses) are shown (pooled data from four independent experiments).

We further assessed the effect of garlic-EO treatment on pro-inflammatory mediator secretion in MLN draining the infected intestines. On day 6 p.i., increased nitric oxide concentrations could be observed in mice from the placebo and garlic-EO cohorts (*p* < 0.001 versus naive), but with a trend towards lower concentrations in the latter versus the former (n.s. due to high standard deviations; Figure 7A). *C. jejuni* induced IFN-γ secretion was enhanced in MLN derived from the placebo (*p* < 0.01; Figure 7B), but not the garlic-EO group. Hence, garlic-EO treatment could dampen *C. jejuni* induced pro-inflammatory mediator secretion in distinct parts of the intestinal tract.

### 3.6. Extra-Intestinal Nitric Oxide Secretion upon Garlic-EO Treatment of C. jejuni Infected IL-10^-/-^ Mice

**Figure 8:**
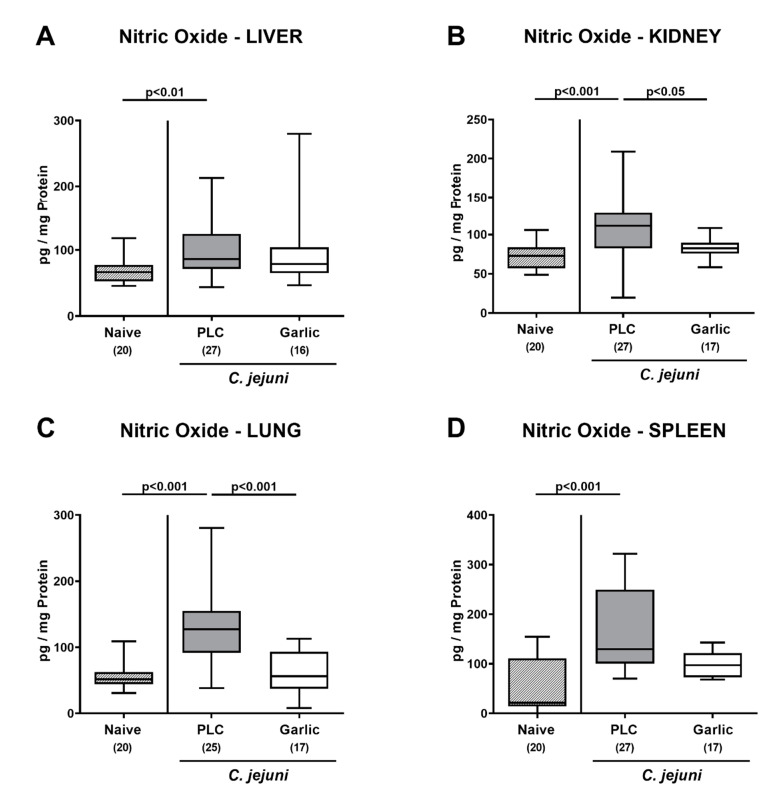
Extra-intestinal nitric oxide secretion upon garlic essential oil treatment of *C. jejuni* infected IL-10^-/-^ mice. Secondary abiotic IL-10^-/-^ mice were orally challenged with *C. jejuni* strain 81-176 on days 0 and 1 followed by treatment with either garlic essential oil (white boxes) or placebo (PLC, gray boxes) via the drinking water starting on day 2 post-infection. Nitric oxide concentrations were determined in culture supernatants of explants taken from the (**A**) liver, (**B**) kidney, (**C**) lung and (**D**) spleen on day 6 post-infection. Naive mice (hatched boxes) served as non-infected and untreated cohort. Box plots (indicating the 25th and the 75th percentiles), whiskers (indicating maximum and minimum values), medians (black bar inside box), significance levels (*p* values) determined by the Kruskal-Wallis test and Dunn’s post-correction and the total number of mice (in parentheses) are shown (pooled data from four independent experiments).

**Figure 9:**
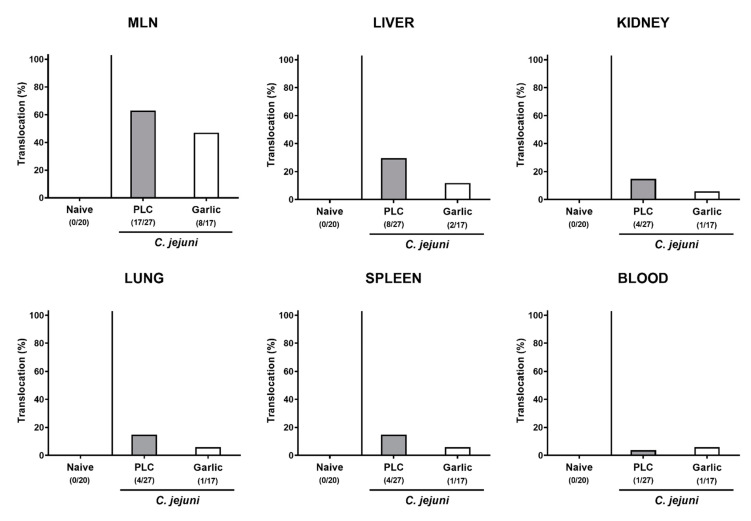
Bacterial translocation upon garlic essential oil treatment of *C. jejuni* infected IL-10^-/-^ mice. Secondary abiotic IL-10^-/-^ mice were orally challenged with *C. jejuni* strain 81–176 on days 0 and 1 followed by treatment with either garlic essential oil (white bars) or placebo (PLC, gray bars) via the drinking water starting on day 2 post-infection. Bacterial translocation frequencies (in %) were determined in explants taken from the mesenteric lymph nodes (MLN), liver, kidney, lung, and spleen and in cardiac blood (as indicated) on day 6 post-infection by culture. Naive mice (hatched bars) served as non-infected and untreated cohort. Bars indicating the translocation frequencies (in %) and the numbers of culture-positive out of the total number of analyzed samples (in parentheses) are shown (pooled data from four independent experiments).

We then tested whether treatment of *C. jejuni* infected mice with garlic-EO might impact pro-inflammatory mediator secretion in extra-intestinal organs. In fact, increased nitric oxide concentrations were measured in the liver, the kidneys, the lungs and the spleen of placebo, but not garlic-EO treated mice on day 6 p.i. (*p* < 0.01–0.001 versus naive; Figure 8). We further assessed the translocation of viable *C. jejuni* cells from the infected intestines to extra-intestinal tissue sites. Our cultural analyses revealed that *C. jejuni* could be isolated less frequently from the MLN (47.1% versus 63.0%), the liver (11.8% versus 29.6%), the kidneys (5.9% versus 14.8%), the lungs (5.9% versus 14.8%) and the spleen (5.9% versus 14.8%) of garlic-EO as compared to placebo treated mice on day 6 p.i. (Figure 9). Of note, *C. jejuni* could be isolated from cardiac blood in single cases only (Figure 9). Hence, garlic-EO treatment resulted in dampened extra-intestinal pro-inflammatory mediator secretion and bacterial translocation during acute murine campylobacteriosis.

## 4. Discussion

Our actual preclinical intervention study provides first evidence for disease-alleviating effects of garlic-EO in acute murine campylobacteriosis. When compared to placebo application, garlic-EO treatment of *C. jejuni* infected mice resulted in (i.) alleviated clinical signs, (ii.) lower ileal *C. jejuni* burdens, (iii.) diminished colonic epithelial cell apoptosis, (iv.) less pronounced pathogen-induced immune cell responses in the large intestines, (v.) dampened pro-inflammatory mediator secretion in intestinal and extra-intestinal compartments, and in (vi.) less frequent translocation of viable *C. jejuni* from the infected and inflamed intestines to organs beyond the gastrointestinal tract.

Garlic-EO treatment could effectively alleviate *C. jejuni* induced clinical signs such as bloody diarrhea and wasting symptoms indicative for systemic inflammatory disease. Since ancient times, garlic has been used to treat a plethora of gastrointestinal morbidities including diarrhea of different etiologies [14,16,43]. When applied to weaned pigs, garlic could alleviate diarrhea upon pathogenic *Escherichia coli* infection [44], for instance, whereas aged garlic extract pretreatment improved methotrexate-induced small intestinal disease including diarrheal symptoms in rats [45].

Whereas on day 6 p.i., the severity of *C. jejuni*-induced histopathological changes in the large intestines did not significantly differ between both treatment cohorts, less pronounced apoptotic cell responses in colonic epithelia could be observed following garlic-EO as compared to placebo treatment. In support, potent anti-apoptotic effects of garlic have been demonstrated to be due to inhibition of oxidative stress pathways [46]. To date, improved clinical and microscopic outcomes upon treatment with different garlic compounds have been shown in various acute intestinal inflammation models. For instance, garlic oil application could ameliorate acute colitis induced by dextran sulfate sodium (DSS) in rats [47], which also held true for water-soluble garlic polysaccharide in acute murine DSS colitis [48], and for prophyl-propane thiosulfonate isolated from garlic in both, DSS- and 2,4-dinitrobenzene sulfonic acid-induced acute colitis in mice [49].

When assessing potential anti-pathogenic effects of garlic-EO in our present study, oral treatment with the natural compound lowered the median *C. jejuni* numbers in the ileum of mice by approximately 1.5 log orders of magnitude within less than a week p.i., whereas a pathogen-lowering effect could not be observed in other parts of the gastrointestinal tract such as the stomach, the duodenum, and the colon. Particularly in the latter, the median pathogen burdens were relatively high with approximately 10^9^ CFU per g luminal content. In the literature, the observed anti-*C. jejuni* effects of garlic in vivo are rather discouraging. For instance, the intestinal *C. jejuni* loads remained unchanged in broiler chickens following a diet containing the garlic derivative propyl-propane thiosulfonate [50]. In support, application of allicin to the drinking water did not affect *C. jejuni* colonization in broiler flock despite promising anti-*C. jejuni* directed results derived from in vitro experiments upon allicin or garlic oil extract co-incubation [51]. Furthermore, garlic extract as feed additive did not prevent colonization of chicken with *C. jejuni* [52]. Hence, the observed improved clinical and microscopic outcomes upon garlic-EO application may not be attributed to the modest lowering in ileal pathogen counts. More importantly, pronounced immune-modulatory effects could be observed upon garlic-EO treatment of *C. jejuni* infected mice, since pathogen-induced increases in large intestinal innate and adaptive immune cell populations such as macrophages/monocytes and T lymphocytes, respectively, were lower in garlic-EO as compared to placebo treated mice on day 6 p.i. These dampened immune cell responses upon garlic-EO treatment were accompanied by less distinct secretion of pro-inflammatory mediators such as nitric oxide, IL-6, MCP-1, TNF-α, or IFN-γ in distinct intestinal compartments including the colon, ileum or MLN. In support, several in vitro studies revealed that garlic and its derivatives suppressed production of pro-inflammatory cytokines such as IL-6, MCP-1, TNF-α, and IFN-γ [53,54,55,56,57], whereas garlic oil was shown to down-regulate pro-inflammatory T helper cell 1 (Th1) responses in rats [26]. Furthermore, pre-incubation of lipopolysaccharide (LPS) stimulated adipocytes with the garlic compound alliin could prevent increases in IL-6 and MCP-1 concentrations [58]. Moreover, in skeletal muscle, garlic oil lowered nitric oxide concentrations during hypoglycemia [59].

Remarkably, the immune-modulatory and the anti-oxidant effects of garlic-EO in particular were not restricted to the intestines of *C. jejuni* infected mice but were additionally effective in extra-intestinal tissue sites as indicated by increased nitric oxide concentrations measured in liver, kidneys, lungs, and spleen derived from placebo control animals as opposed to garlic-EO treated mice that did not differ from basal levels. These results are supported by previous studies reporting anti-oxidant effects of garlic aqueous extract in liver, kidney and brain during lead-induced oxidative stress in rats [19], whereas allicin protected mice from fatty liver disease by improving anti-oxidant and anti-inflammatory functions [60].

A key mechanism of *C. jejuni*-induced immunopathology during acute campylobacteriosis in infected secondary abiotic IL-10^-/-^ mice is the induction of the TLR-4 dependent signaling cascade by bacterial cell wall-derived LOS [4,32]. Importantly, garlic was shown to suppress LPS-induced TLR-4 dimerization [61]. Hence, with its anti-TLR-4 directed effect garlic does in fact impact a key event in *C. jejuni*-induced immunopathology.

In addition, our study revealed that in garlic-EO treated mice less frequent translocation of viable *C. jejuni* from the inflamed intestines to extra-intestinal organs could be observed when compared to placebo controls. Even though not directly addressed in our present survey, but these observations may point towards a less compromised intestinal epithelial barrier function upon garlic-EO versus placebo application during acute murine campylobacteriosis.

Given the short period of garlic-EO treatment, namely four days from day 2 until day 6 p.i., the disease-alleviating effects of garlic in the here applied acute murine campylobacteriosis model may be considered as relatively potent. It is tempting to speculate that the disease-alleviating effects of garlic-EO may be even more pronounced upon prophylactic treatment. In future studies we will therefore assess the *C. jejuni*-induced disease outcome when initiating the oral application of the natural compound prior infection.

According to the US Food and Drug Administration, the human use of garlic-EO can be considered as generally safe. However, the intake of excessive amounts of garlic is known to cause gastric irritations in sensitive individuals [16]. Considering that the median 50% lethal dose (LD_50_) of garlic-EO for rats is indicated with 1360 mg/kg body weight [62], the here applied daily oral garlic-EO dose of 200 mg/kg is far below. Besides the short application period of four days only, one needs further to take into consideration that upon enteral reabsorption the systemic concentrations of biologically active garlic-EO derived metabolites are even lower and make adverse reactions highly unlikely.

## 5. Conclusions

Our preclinical intervention study provides first evidence that garlic-EO exerts a multitude of orchestrated immune-modulatory effects during acute *C. jejuni* infection of mice and might therefore be considered as a potent disease-alleviating and safe treatment option for acute campylobacteriosis in humans. Given that essential oils are known to interact with the bacterial cell membrane leading to cell lysis and death [63], natural compounds including garlic-EO may additionally constitute promising adjunct antibiotic-independent options to enhance bioavailability of common antibiotics or even exert synergistic effects resulting in improved efficacy in the combat of campylobacteriosis caused by multi-drug resistant *Campylobacter* strains [64]. This needs to be explored in future studies, however.

## Data Availability

The data presented in this study are available on request from the corresponding author.
